# wideband high-gain low-profile series-fed antenna integrated with optimized metamaterials for 5G millimeter wave applications

**DOI:** 10.1038/s41598-023-50769-y

**Published:** 2024-01-02

**Authors:** Bashar A. F. Esmail, Slawomir Koziel, Anna Pietrenko-Dabrowska, Dustin Isleifson

**Affiliations:** 1 Department of Engineering, Reykjavik University, 102 Reykjavík, Iceland; 2 Faculty of Electronics, Telecommunications and Informatics, Gdansk University of Technology, 80-233 Gdansk, Poland; 3 Department of Electrical & Computer Engineering, University of Manitoba, Winnipeg, MB R3T 5V6 Canada

**Keywords:** Electrical and electronic engineering, Computational science

## Abstract

This paper presents a series-fed four-dipole antenna with a broad bandwidth, high gain, and compact size for 5G millimeter wave (mm-wave) applications. The single dipole antenna provides a maximum gain of 6.2 dBi within its operational bandwidth, which ranges from 25.2 to 32.8 GHz. The proposed approach to enhance both gain and bandwidth involves a series-fed antenna design. It comprises four dipoles with varying lengths, and a truncated ground plane. These dipoles are connected in series on both sides, running in parallel through a microstrip line. The proposed design significantly enhances the bandwidth, which extends from 26.5 to 40 GHz. This frequency range effectively covers the 5G bands of 28 and 38 GHz. The expedited trust-region (TR) gradient-based search algorithm is utilized to optimize the dimensions of the antenna components, resulting in a maximum gain of 11.2 dBi at 38 GHz. To further enhance the gain, modified H-shaped metamaterial (MTM)-based unit cells are integrated into the antenna substrate. The TR algorithm is employed once more to optimize the MTM dimensions, yielding a maximum gain of 15.1 dBi at 38 GHz. The developed system is experimentally validated, showing excellent agreement between the simulated and measured data.

## Introduction

The increasing need for wide bandwidth and high data rates to support numerous users is the main driving force behind the development of fifth-generation (5G) technology. To address the aforementioned demands, the use of millimeter-wave spectrum (mm-wave) appears to be a promising solution that can significantly improve the data rates and capacity of 5G networks^1^. Several mm-wave bands, such as 28 GHz, 38 GHz, and 60 GHz, are being considered for deployment to support 5G wireless communications^2^. Extensive research is being carried out on the 28 GHz and 38 GHz bands for 5G networks, which can considerably enhance the network's performance compared to the low frequencies of 4G^3^. Nonetheless, the mm-wave spectrum encounters significant propagation losses that curtail its coverage area. High-gain antennas can mitigate this issue, and various techniques have been reported in scientific literature to enhance mm-wave antenna gain. These include the use of multiple substrates^4^, multiple shorting pins^5^, dielectric lenses^6^, and artificial materials^7,8^. However, these methods result in large profile structures, complex power distribution, fabrication complexities, and narrow bandwidth. Series-fed antennas have been proposed in the literature to boost the gain performance [9−12]. The authors of Ref.^9^ proposed an eight-element dipole with overall dimensions of 10 × 36.5 mm^2^ to attain a maximum gain of 10.9 dBi at 28 GHz. Nevertheless, even with the use of eight elements and a notably large size, the gain in this design is considered to be low. A series-fed antenna with microstrip-to-grounded co-planar waveguide (CPW) transition was presented in Ref.^10^ to achieve a maximum gain of 10.3 dBi in the range 27.25−28.5 GHz. Yet, with dimensions of 22.8 × 67 mm^2^, the antenna is considered bulky when operating in the high-frequency range. In Ref.^11^, an eight-element series-fed dipole antenna based on substrate integrated waveguide (SIW) was introduced to attain a maximum gain of 12.3 dBi within the 21.1–27.82 GHz frequency range. The physical dimensions of the antenna were considerably large, measuring 10 × 59 mm^2^, despite its good gain performance. The four-element dipole array presented in Ref.^12^ achieved a wide bandwidth of 1.81−3.78 GHz and a maximum gain of 6.78 dBi. The antenna's physical size of 64 × 105 mm^2^ is considerably large, alongside its low gain.

High gain is often achieved by using high-profile structures or array antennas since the gain is directly proportional to the antenna's aperture. However, there is a growing demand for compact devices featuring small physical size, which poses a challenge in the context of gain enhancement. Over the past decade, metamaterials (MTMs) have emerged as a cost-effective way to improve gain without substantially increasing the antenna's profile^13^. MTMs are a remarkable type of artificial materials that exhibit unique characteristics not observed in natural materials. Integrating these composite structures into various antennas can significantly improve their gain performance [14 − 16]. The authors of Ref.^14^ employed seven layers of MTMs, with six air gaps above the patch antenna, to achieve a gain of 13.6 dBi at 28 GHz. Similarly, in Ref.^15^, the authors added one layer of different MTM structures over the patch antenna to attain a maximum gain of 11.59 dBi at 28 GHz. However, the incorporation of MTM layers leads to the increased size and complexity of the design, which form a 3D structure. The MTM-based dual bow-tie antenna with high gain and low profile was proposed to operate at the mm-wave spectrum^16^. The antenna is designed on a single-layer Rogers PCB with a high gain of 12.2 dBi at 29 GHz.

In the light of the mentioned literature, none of the stated antennas discussed incorporating the series-fed antenna with MTMs and employment of the optimization techniques to attain high gain at the mm-wave spectrum. The use of rigorous optimization methods is instrumental in effectively managing multiple geometry parameters and design objectives, which cannot be accomplished through conventional methods such as parametric studies based on engineering experience. This paper addresses the design and optimization of a high-gain antenna for broadband mm-wave applications. Two steps of optimization are employed to improve the basic antenna and the MTM antenna performance. The first stage is implemented by optimizing the series-fed antenna parameters to achieve high gain, followed by the design of MTM and integrating it with an optimized series-fed antenna to enhance the gain further. The technical contributions and originality of this work can be summarized as follows:

I. Design of a low-profile, four-element series-fed dipole antenna to achieve high gain, reaching 11.2 dBi at 38 GHz, and a wide bandwidth in the range of 26.5 − 40 GHz;
II. Design an MTM structure and integrate it with the antenna to further enhance the gain, achieving a maximum of 15.1 dBi at 38 GHz while maintaining a low profile and low geometrical complexity;
III. Development and execution of a customized two-stage optimization approach to enhance the gain of the series-fed antenna, and MTM antenna. The accelerated trust-region gradient-based search algorithm with restricted sensitivity updating strategy is employed to optimize the geometrical dimensional of the basic antenna and the MTM antenna while maintaining a low profile and simplicity. For that purpose, a regularization-based objective function is defined, which enables simultaneous control over the antenna gain and its reflection response.

Compared to the state-of-the-art developments reported in the literature, the proposed design provides a low computational cost, low-profile, and lower-complexity system with high gain and broad bandwidth. The remaining part of the work is organized as follows. Section “Series-fed antenna design” introduces the series-fed dipole antenna. Section “Antenna performance optimization” elaborates on series-fed antenna performance optimization. In Sect. “MTM for antenna gain enhancement”, the MTM for antenna gain enhancement is presented. Optimization of the antenna ‘s MTMs is discussed in Sect. “MTM antenna gain performance optimization”. The experimental results are discussed in Sect. “Experimental results and discussion”. Section “Conclusion” concludes the paper.

## Series-fed antenna design

**Figure 1:**
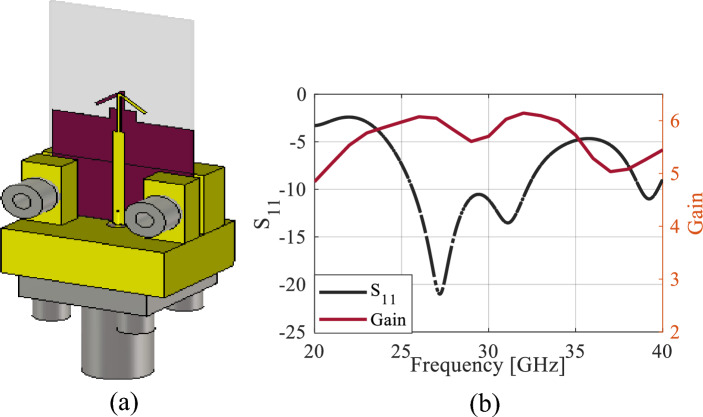
Single-element antenna: (**a**) configuration, (**b**) reflection coefficient and realized gain.

The single-element dipole configuration is shown in Fig. 1a. It consists of tilted dipole arms implemented on both sides of the substrate, and a truncated ground plane. By adopting a tilted dipole orientation, the size of antennas can be reduced. Additionally, such antennas exhibit better radiation characteristics than their straight-dipole counterparts^9,17^. The antenna is designed on a 0.508 mm-thick Rogers RT5880 substrate (dielectric constant* ε*_*r*_ = 2.2, tangent loss tan(*δ*) = 0.0009). The electromagnetic wave transmitted to the microstrip line was conveyed to the dipole using a parallel stripline printed on both sides of the substrate. Figure 1b depicts the antenna reflection coefficient and the gain. The 0.5λ mode (the dipole length is approximately half a wavelength), resonating at 28 GHz, represents the fundamental and most frequently employed mode. The results indicate that the antenna has a wide bandwidth of 7.6 GHz (25.2−32.8 GHz), making it suitable for covering the 5G band of 28 GHz. However, the maximum gain of 6.2 dBi within the operational bandwidth is relatively low for mm-wave communications. The demand for wide bandwidth and high-gain antennas is increasing to overcome path loss challenges encountered in the mm-wave spectrum. To address this issue, two stages of gain enhancement are utilized, involving multiple series elements and embedded MTMs, which significantly boost the antenna's gain.

**Figure 2:**
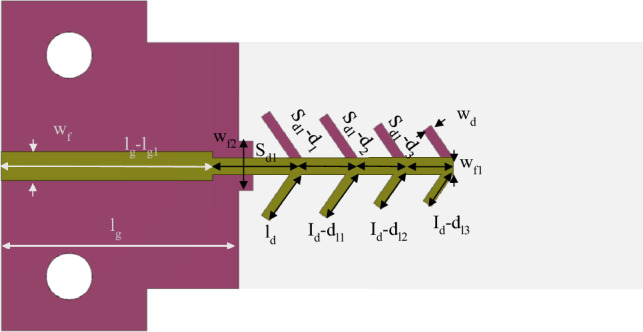
Proposed series-fed antenna configuration. Dimensions upon accomplishing the optimization process (all in millimeters except *angle*, which is in degrees): S_*d1*_ = 2.66, *w*_*f*_ = 1.18, *w*_*f*1_ = 0.70, *l*_*g*_ = 9.60,* l*_*g*1_ = 1.05,* w*_*l*_ = 1.65, *angle* = 34.58,* l*_*d*_ = 2.52*, w*_*d*_ = 0.40*, d*_1_ = 0.40*, d*_2_ = 0.71*, d*_3_ = 0.72*, d*_*l*1_ = 0.27*, d*_*l*2_ = 0.63,* d*_*l*3_ = 0.75, and *w*_*f*2_ = 2.0.

**Figure 3:**
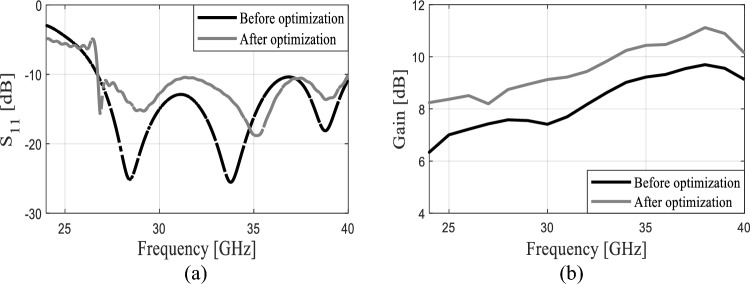
The series-fed antenna performance before and after the optimization; (**a**) the reflection coefficient and (**b**) the realized gain.

The design of the four-element dipole antenna is shown in Fig. 2. In this design, the length and spacing of the dipoles were optimized to enhance the gain and bandwidth. Before the optimization process, the length and spacing of the four-element dipole were uniformly reduced by 0.2 mm each. Subsequently, non-uniform reductions were implemented using the optimization method to achieve high gain and broad bandwidth. The reflection coefficients of the four-element dipole before and after the optimization are shown in Fig. 3a. The incorporation of four dipoles exerts a notable influence on the reflection coefficient performance, resulting in a significantly broader bandwidth from 26.5 to 40 GHz. This enhancement represents a substantial improvement compared to the limited range of the single dipole, which spans from 25.2 to 32.8 GHz. Furthermore, after the optimization process, the antenna continues to cover the same frequency band, which has been enforced through appropriate formulation of the design optimization task and the objective function. The results indicate that the series-fed antenna has a broad bandwidth, making it suitable for covering the 5G bands of 28 and 38 GHz. Conversely, the gain of the single dipole antenna, which reaches a maximum of 6.2 dBi at 38 GHz, is considered inadequate for mm-wave communications. In Fig. 3b, the gains of the series-fed antenna are depicted both before and after the optimization process. It is evident that the series-fed antenna demonstrates significantly improved gain across the entire operating band when compared to the single dipole antenna (cf. Fig. 1b), even before any optimization. This suggests that further enhancements can be achieved through appropriate design optimization using formal numerical techniques. The TR gradient-based search procedure is utilized to optimize the length and spacing of the four dipoles, aiming to achieve a maximum gain of 11.2 dBi at 38 GHz. The optimization process has been outlined in Section “Antenna performance optimization”.

## Antenna performance optimization

### Problem formulation

We adopt the following notation. Design variables of the antenna under design (normally, geometry parameters) will be denoted as a vector ***x*** = [*x*_1_ … *x*_*n*_]^*T*^; *f* will denote frequency. Accordingly, *S*_11_(***x***,*f*) and *G*(***x***,*f*) will stand for antenna reflection coefficient and end-fire gain at design ***x*** and frequency *f*.

There are two objectives of the antenna design optimization process, which are to be achieved simultaneously:

- Improve the antenna end-fire gain *G*(***x***,*f*) over the frequency range frequency range 26.5 GHz ≤ *f* ≤ 40 GHz;
- Ensure sufficient impedance matching, so that the following condition is satisfied, |*S*_11_(***x***,*f*)|≤ –10 dB for all *f* ∈ *F* = [26.5 40.0] GHz.

Now, gain maximization may be understood in different ways, e.g., improvement of the maximum in-band gain. In our case, due to a broad range of operating frequency, we are interested in improving the average in-band gain, which is generally more advantageous because normally leads to enhancement of this parameter over the entire range of frequencies of interest. The average gain is defined as

1 $${G}_{A}\left(x\right)=\frac{1}{{f}_{max}-{f}_{min} }{\int }_{{f}_{min}}^{{f}_{max}}G\left(x,f\right)df.$$

Here, *f*_min_ = 26.5 GHz, and *f*_max_ = 40 GHz.

The quality of design ***x*** is measured using a scalar merit function *U*, which is defined to account for both the primary objective (average gain maximization), and reflection constraint. We use the following formulation

2 $$U\left(x\right)=-{G}_{A}\left(x\right)+\beta {\left[\frac{{\text{max}}\left\{S\left(x\right)+\mathrm{10,0}\right\}}{10}\right]}^{2},$$

where

3 $$S\left(x\right)=\mathit{max}\{f\in [{f}_{min} {f}_{max}]:\left|{S}_{11}\left(x,f\right)\right|\},$$

is the maximum in-band reflection. The second term in (2) is a penalty factor that quantifies relative violation of the condition |*S*_11_(***x***,*f*)|≤ –10 dB, and increases the value of the merit function using a proportionality coefficient *β*. The latter is set to 10^2^, so that violations at the level of a fraction of decibel lead to noticeable changes of *U*(***x***)^18^. Higher violations result in increasing the function (2), i.e., the presence of the penalty factor effectively enforces constraint satisfaction. This mechanism is referred to as implicit constraint handling^19^. It is recommended for treating computationally expensive constraints, and our reflection condition belongs to this category (EM simulation of the antenna is required to for its evaluation).

At this point, we are ready to formulate the antenna optimization task. As discussed in the previous paragraph, better designs, i.e., those associated with higher gain and being feasible (with respect to the constraint) correspond to lower values of *U*(***x***), i.e., seeking for the optimum is equivalent to reducing *U*(***x***). Consequently, the optimum design x^*^ can be found by solving a nonlinear minimization problem.

4 $${{\varvec{x}}}^{*}={\text{arg}}\underset{\boldsymbol{x }\in {\varvec{X}}}{{\text{min}}}U({\varvec{x}}).$$

### Optimization algorithm

The problem (4) is solved here using the trust-region (TR) gradient-based algorithm^20^ with numerical derivatives. The antenna response gradients (in particular, the Jacobian matrix) are evaluated through finite differentiation^21^. The operating principles of the TR procedure involve yielding a series ***x***^(*i*)^, *i* = 0, 1, …, of approximate solutions to (4) by solving a sub-problem

5 $${\boldsymbol{x}}^{(i + 1)} = \mathop {\max }\limits_{\substack{ {\boldsymbol{x}} \in X \\ ||{\boldsymbol{x}} - {\boldsymbol{x}}^{(i)} || \le d^{(i)} } } U_{L}^{(i)} ({\boldsymbol{x}}).$$

The objective function *U*_*L*_^(*i*)^ is analytically identical to the function *U* of (2); however, it is evaluated using first-order linear approximation models of antenna responses. The latter are defined as

6 $${{\varvec{L}}}_{{S}_{11}}^{\left(i\right)}\left({\varvec{x}}, f\right)= {S}_{11}\left({\varvec{x}}, f\right)+ \nabla {{\varvec{S}}}_{11}\left({{\varvec{x}}}^{\left(i\right)}, f\right)\cdot \left({\varvec{x}}- {{\varvec{x}}}^{\left(i\right)}\right),$$

and

7 $${{\varvec{L}}}_{G}^{\left(i\right)}\left({\varvec{x}}, f\right)= G\left({\varvec{x}}, f\right)+ \nabla {\varvec{G}}\left({{\varvec{x}}}^{\left(i\right)}, f\right)\cdot \left({\varvec{x}}- {{\varvec{x}}}^{\left(i\right)}\right),$$

for reflection and gain characteristics, respectively. Therein, ∇*S*_11_(***x***^(*i*)^,*f*) and ∇*G*(***x***^(*i*)^,*f*) are gradients of *S*_11_ and *G*, respectively, at ***x***^(*i*)^ and *f*. Using this notation, the function *U*_*L*_^(*i*)^ takes the form of

8 $${{\varvec{U}}}_{L}^{\left(i\right)}\left({\varvec{x}}\right)=-\frac{1}{{f}_{max}-{f}_{min} }{\int }_{{f}_{min}}^{{f}_{max}}{{\varvec{L}}}_{G}^{\left(i\right)}\left(x, f\right)df+\beta {\left[\frac{{\text{max}}\{{\text{max}}\{f\in [{f}_{min} {f}_{max}]:\left|{{\varvec{L}}}_{{S}_{11}}^{\left(i\right)}\left(x, f\right)\right|\}+10\}}{10}\right]}^{2}$$

As indicated in (5), the function *U*_*L*_^(*i*)^ is locally optimized in the region of radius *d*^(*i*)^, which is adaptively adjusted using standard TR rules (see, e.g., Ref.^20^). The algorithm termination is based upon convergence in argument (||***x***^(*i*+1)^ – ***x***^(*i*)^||< *ε*; here, *ε* = 10^–3^) or sufficient reduction of the search radius (*d*^(*i*)^ < *ε*).

A major bottleneck of EM-driven antenna optimization is its high computational cost, as each iteration of the TR algorithm involves at least *n* + 1 antenna evaluations (*n* being the number of geometry parameters). The number of iterations typically grows with the problem dimensionality, so that the computational cost of the search process scales as *n*^*α*^, where *α* is usually between 1.5 and 2.0. For *n* > 10, and EM simulation time between 15 to 30 min (which is the case for the proposed antenna), the running time of the optimization process may readily exceed several days.

In this work, we use an accelerated version of the TR algorithm^22^, which leverages selective employment of a rank-one Broyden formula (BF)^23^. In this method, finite differentiation is replaced by updating the Jacobian matrix using BF, which is applied to directions, which are sufficiently well aligned with the most recent design relocation. We define the alignment factors

9 $$\gamma_{k}^{\left( i \right)} = \, \left| {{\boldsymbol{h}}^{\left( i \right)T} {\boldsymbol{e}}^{(k)} } \right|/\left| {\left| {{\boldsymbol{h}}^{(i)} } \right|} \right|,\,\,k\, = \,{1},\, \ldots ,\,n,$$

where ***e***^(*k*)^ are the standard basis vectors (i.e., ***e***^(*k*)^ = [0 … 1 … 0]^*T*^, with one on the *k*-th position), and ***h***^(*i*+1)^ = ***x***^(*i*+1)^ – ***x***^(*i*)^. For any index *k*, if *γ*_*k*_^(*i*)^ is larger than a user-defined threshold *γ*_min_, the *k*th row of Jacobian is updated using BF. The control parameter 0 ≤ *γ*_min_ ≤ 1 is used to control the trade-offs between the computational savings and the design quality. As recommended in Ref.^22^, *γ*_min_ = 0.9 is in this work. The algorithm^22^ typically allows for up to fifty percent reduction of the optimization cost for lower-dimensional problems, and over sixty percent savings for *n* > 10, with minor degradation of the design quality.

### Optimization of basic antenna

Optimization of the proposed antenna is arranged into two stages. The first stage is parameter tuning of the basic antenna (without MTM). The design objectives are as explained in subsection A. The structure is described by fourteen parameters ***x*** = [*w*_*f*_* w*_*f*1_ *l*_*g*_* l*_*g*1_* w*_*l*_* angle l*_*d*_* w*_*d*_* d*_1_* d*_2_* d*_3_* d*_*l*1_* d*_*l*2_* d*_*l*3_]^*T*^. The parameter space *X* is defined using the lower and upper bounds on design variables as follows (all dimensions in millimeters except *angle*, which is in degrees): 0.8 ≤ *w*_*f*_ ≤ 1.2, 0.2 ≤ *w*_*f*1_ ≤ 0.8, 8 ≤ *l*_*g*_ ≤ 11, 0.2 ≤ *l*_*g*1_ ≤ 1.1, 1.5 ≤ *w*_*l*_ ≤ 2.4, 15 ≤ *angle* ≤ 40, 2.2 ≤ *l*_*d*_ ≤ 2.8, 0.2 ≤ *w*_*d*_ ≤ 0.4, 0.2 ≤ *d*_1_, *d*_*l*1_ ≤ 0.5, 0.4 ≤ *d*_2_, *d*_*l*2_ ≤ 0.8, and 0.6 ≤ *d*_3_, *d*_*l*3_ ≤ 1.1. We also have additional constraints imposed to ensure geometrical consistency of the antenna: *w*_*f*_ ≥ *w*_*f*1_, *w*_*l*_ ≤ *s*_*d*1_ − 1.55, *d*_*l*1_ < *d*_*l*2_, *d*_*l*2_ < *d*_*l*3_, *d*_1_ < *d*_2_, and *d*_2_ < *d*_3_.

The initial design ***x***^(0)^ = [0.9 0.4 10.5 0.7 1.9 45 2.7 0.55 0.4 0.65 0.8 0.3 0.75 0.94]^*T*^ has been obtained at the stage of antenna topology development using initial parametric studies. The optimized design, x^*^ = [1.18 0.70 9.60 1.05 1.65 34.58 2.42 0.40 0.40 0.71 0.72 0.27 0.73 0.85]^*T*^, has been obtained using the algorithm outlined in subsection B at the cost of only about one hundred EM analyses of the device.

Figure 2 shows the antenna structure with the optimized dimensions, whereas Fig. 3 illustrates the reflection response and realized end-fire gain at the initial design and after accomplishing the optimization process. As it can be observed, the gain has been significantly enhanced across the entire bandwidth owing to the formulation of the problem (maximization of the average in-band gain). The mean gain increased from 9.8 dBi at the initial design to 11.2 dBi at the final design (both at 38 GHz). At the same time, it should be emphasized that the reflection condition is well-maintained throughout the entire frequency range of interest, i.e., is is lower than − 10 dB between 25.6 GHz and 40 GHz as shown in Fig. 3a.

## MTM for antenna gain enhancement

**Figure 4:**
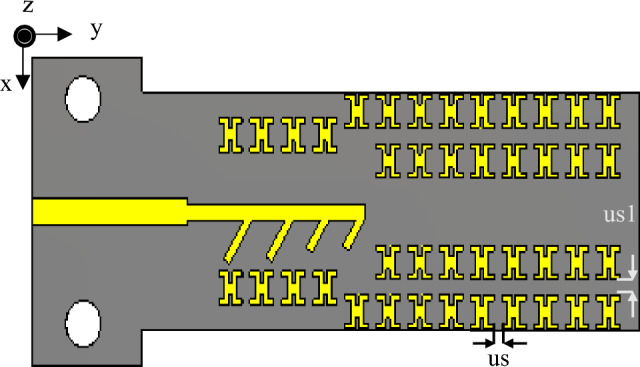
The proposed series-fed antenna configuration with MTM loading.

**Figure 5:**
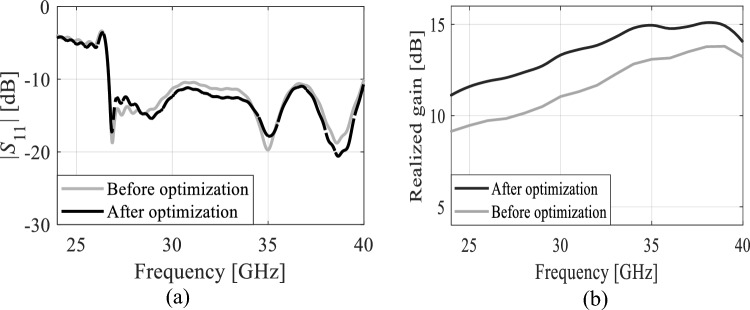
MTM antenna performance before and after the optimization; (**a**) reflection coefficients, (**b**) realized gain.

**Figure 6:**
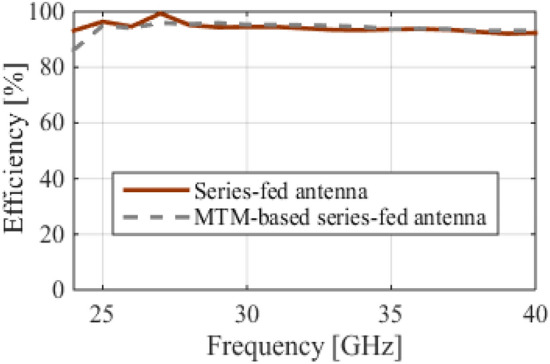
The radiation efficiency of the series-fed four-dipole antenna with and without MTMs.

**Figure 7:**
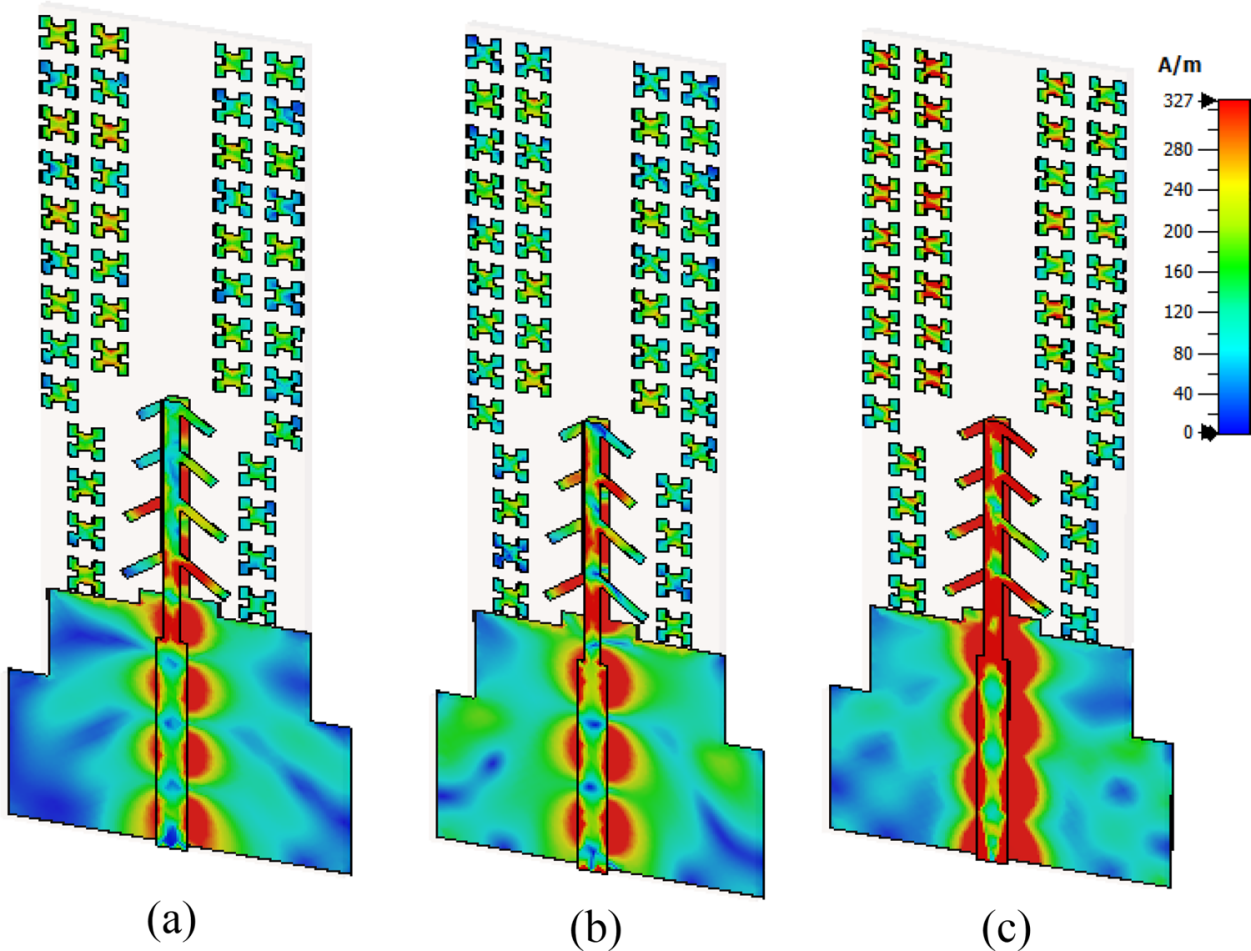
The surface current distribution of the MTM-based series-fed four-dipole antenna at (**a**) 28 GHz, (**b**) 34 GHz, and (**c**) 38 GHz.

To further boost the gain, a set of modified H-shaped MTMs has been embedded on the antenna substrate in the end-fire direction (the *xy*-plane). The antenna configuration with the integrated MTMs is demonstrated in Fig. 4. Through the insertion of MTMs into the antenna substrate and numerical optimization of their geometry parameters, it is feasible to reduce the design cycle, including the alterations to geometry dimensions, while attaining an optimal design. In this context, optimization of the MTMs embedded into the antenna system (rather than designing the structure separately and then integrating it into the antenna substrate, which necessitates re-optimizing the number and location of unit cells), allows for saving time and resources, as compared to carrying out these stages separately. The reflection coefficient and gain plots (before and after the optimization) of the series-fed antenna loading MTMs have been presented in Fig. 5a and b, respectively. The inclusion of the MTM minimally affects the impedance matching performance of the MTM antenna in comparison to the unloaded antenna, cf. Fig. 3a. Furthermore, the reflection coefficient before and after the optimization process remains almost the same. Prior to optimization, the MTM antenna exhibits a significantly improved gain, reaching maximum of 13.8 dBi at 38 GHz, as illustrated in Fig. 5b. This implies that formal numerical techniques can be employed for suitable design optimization to achieve further enhancements. The TR algorithm is utilized to optimize the MTM dimensions and attain a maximum gain of 15.1 dBi at 38 GHz, as illustrated in Fig. 5b. The optimization process details can be found in Sect. “MTM Antenna Gain Performance Optimization”. The radiation efficiency of the series-fed four-dipole antenna, with and without MTMs, is illustrated in Fig. 6. Both the antenna and the MTM antenna exhibit stable radiation efficiency, exceeding 94% across the operating bandwidth. This high efficiency is primarily attributed to the utilization of a low-loss substrate^15^. The surface current distributions for the MTM-based series-fed four-dipole antenna are illustrated in Fig. 7 at frequencies of 28 GHz, 34 GHz, and 38 GHz. The long dipole elements, particularly the first two dipoles, demonstrated concentrated current at lower operating frequencies (28 GHz), as depicted in Fig. 7a. Furthermore, at this frequency, the currents concentrate in the MTM unit cells both adjacent to and above the series-fed four-dipole antenna. This concentration assists in directing the electromagnetic waves toward the emission direction, resulting in an improvement in gain. The illustration in Fig. 7b portrays the current distribution of the MTM antenna at 34 GHz. The concentration of the surface current distribution from the first to the third dipole elements is observed at this frequency. Furthermore, the currents are focused in MTM unit cells, both adjacent to and above the series-fed four-dipole antenna, demonstrating behavior almost comparable to that at 28 GHz.In Fig. 7c, the current distribution of the MTM antenna at 38 GHz is depicted. At this frequency, currents concentrate across all dipoles. Additionally, the MTM currents show pronounced concentrations throughout the unit cells, spanning both sets adjacent to and above the developed antenna. Notably, this configuration demonstrates better performance compared to that at 28 GHz and 34 GHz. The antenna achieves its highest gain at this frequency, with the maximum number of dipole elements in operation and almost all MTMs actively engaged.

## MTM antenna gain performance optimization

The second stage of the optimization process is to improve the gain of the MTM-enhanced antenna shown in Fig. 4. At this stage, we use thirteen variables gathered into a vector ***x*** = [*u*_*l*_* u*_*w*_* r*_*p*_* u*_*p*_* c*_*l*_* c*_*w*_* u*_*s*_* u*_*s*1_ *w*_*f*_* w*_*f*1_ *l*_*g*1_ *l*_*d*_* w*_*d*_]^*T*^. Some of these parameters are specific to the MTM, e.g., *u*_*s*_ and *u*_*s*1_ represent the separation between unit cells in the vertical and horizontal directions, cf. Figs. 4 and 7a. The remaining parameters are pertinent to the basic antenna structure, and are re-used at the second optimization stage as additional degrees of freedom to improve the antenna gain. The parameter space *X* is defined as interval [***l u***], where the lower and upper bound vectors are ***l*** = [1.00 1.00 0.20 0.20 0.20 0.20 0.20 0.20 0.8 0.2 0.2 2.2 0.2]^*T*^, and ***u = ***[1.55 1.40 0.40 0.45 0.55 0.40 0.55 0.80 1.2 0.8 1.2 2.6 0.4]^*T*^ (all dimensions in millimeters). We also have additional constraints imposed to ensure geometrical consistency of the MTM: *u*_*w*_ − 2*u*_*p*_ ≥ 0.4 mm, and *u*_*w*_ − *c*_*w*_ ≥ 0.8 mm.

**Figure 8:**
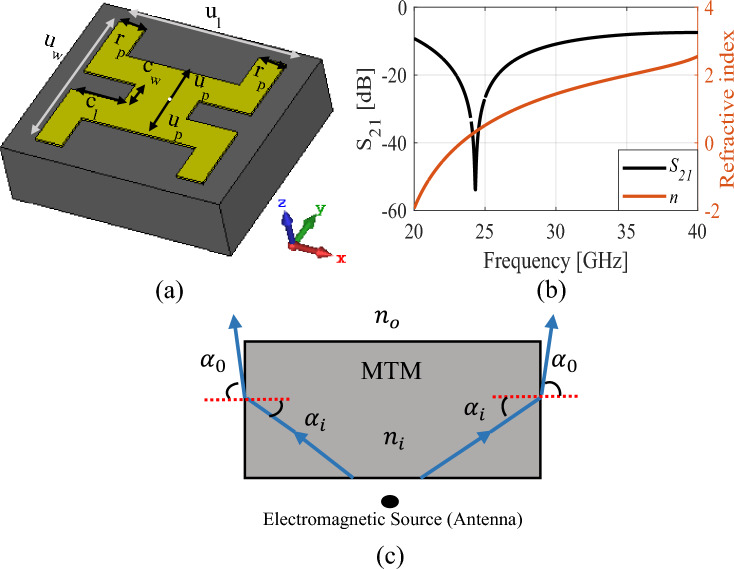
Proposed unit cell geometric specifications and its performance; (**a**) the configuration, dimensions:* u*_*l*_ = 1.45 mm, *u*_*w*_ = 1.30 mm, *r*_*p*_ = 0.23 mm, *u*_*p*_ = 0.34 mm, *c*_*l*_ = 0.45 mm, *c*_*w*_ = 0.2 mm, (**b**) transmission coefficient and the refractive index, and (**c**) gain enhancement mechanism.

The initial design ***x***^(0)^ = [1.36 1.29 0.28 0.31 0.41 0.23 0.39 0.62 1.18 0.70 1.05 2.41 0.40]^*T*^ was obtained through parametric studies, with the exception the last five variables. Those values were inherited from the first optimization stage. The final design x^*^ = [1.45 1.30 0.23 0.34 0.45 0.20 0.44 0.65 1.18 0.70 1.05 2.41 0.40]^*T*^ has been found using the algorithm of subsection B at the cost of less than one hundred EM antenna simulations. As it can be observed, the average in-band gain has been increased considerably by the incorporation of the MTM and the second optimization stage. The average gain in the final design reaches 13.5 dBi, with the maximum gain at the level of about 15.1 dBi at 38 GHz. At the same time, the reflection constraint is well maintained (cf. Fig. 5a). The optimized MTM unit cell is shown in Fig. 8a. To guarantee the desired response, boundary arrangements are assigned to all sides of the structure. The electric (magnetic) conductor boundary is allocated along the *x*(*z*)-direction, whereas the *y*-axis is configured to propagate a normal incident electromagnetic wave. The unit cell transmission coefficient and the refractive index are depicted in Fig. 8b. The unit cell exhibits a refractive index greater than one across the 26.5–40 GHz frequency range. Snell’s law of refraction can explain the mechanism of gain enhancement, which is expressed as $sin{\alpha }_{i}\cdot {n}_{i }=sin{\alpha }_{0}\cdot {n}_{0 },\,\mathrm{ where}$ *n*_*i*_*(α*_*i*_*)* and *n*_0_*(α*_0_*)* are the refractive indices (angles) of the MTM and air^24^. The wave propagation in both MTM and air has been illustrated in Fig. 8c. When maintaining a fixed incident angle *α*_*i*_, a higher refractive index *n*_*i*_ results in a larger refractive angle *α*_0_. To achieve this characteristic, the proposed MTM refractive index should be greater than one (*n*_*MTM*_ > 1) at the desired frequency. Consequently, the energy passing through the MTM structure converges and focuses in the direction of emission.

## Experimental results and discussion

**Figure 9:**
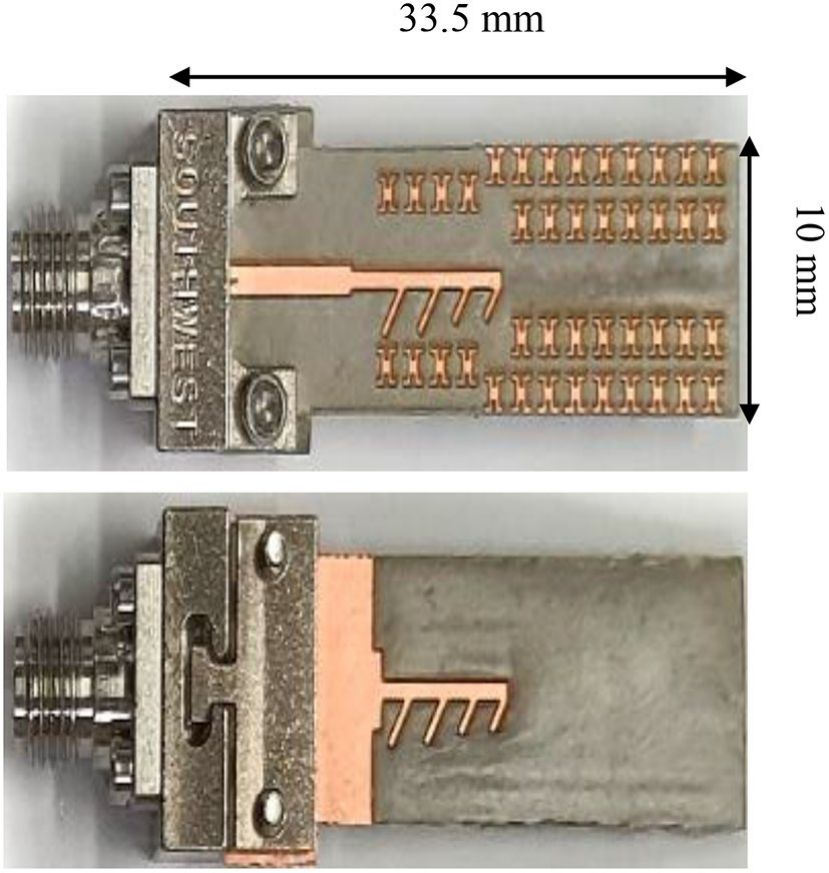
The fabricated front and back views of the MTM-based antenna.

**Figure 10:**
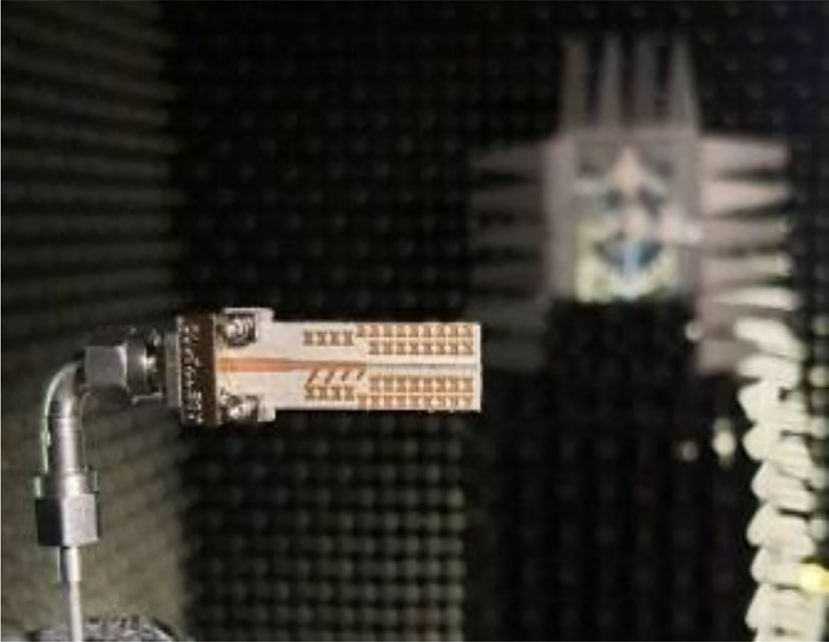
The radiation pattern measurement setup.

**Figure 11:**
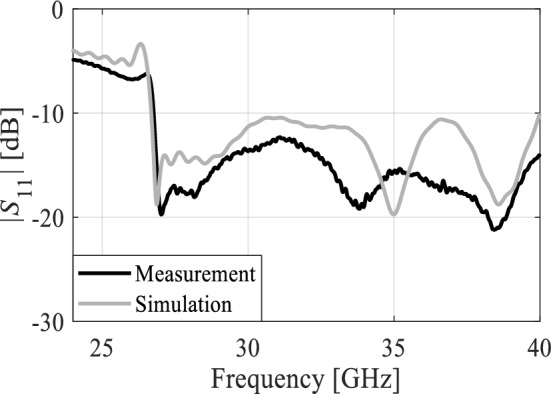
The simulated and measured S-parameters of the MTM antenna.

**Figure 12:**
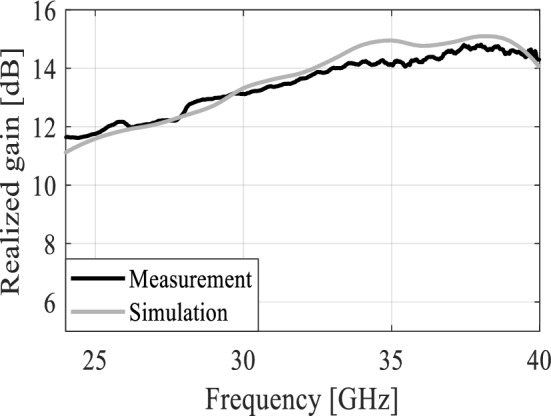
The simulated and measured gain.

**Figure 13:**
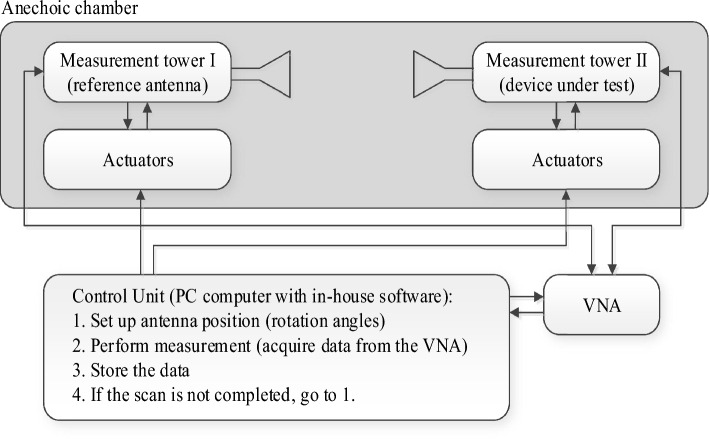
Block diagram of the far-field measurement procedure.

**Figure 14:**
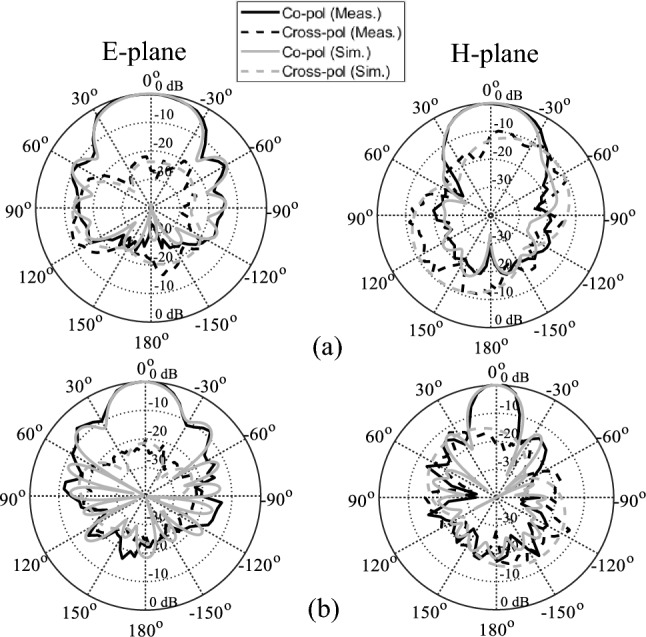
The simulated and measured radiation patterns of the MTM antenna at (**a**) 28 GHz, and (**b**) 38 GHz.

The MTM-based series-fed antenna, optimized using formal numerical techniques, has been manufactured to confirm the simulation results and demonstrate its suitability for 5G mm-wave applications. The antenna prototype is shown in Fig. 9. The reflection coefficient was measured using an Anritsu vector network analyzer MS4644B (0–40 GHz), whereas the radiation patterns were verified in an anechoic chamber, as shown in Fig. 10. The comparison between the simulated and measured |*S*_11_| is presented in Fig. 11. The developed antenna offers a broad impedance bandwidth spanning from 26.5 to 40 GHz, which effectively covers the 5G bands at 28 GHz and 38 GHz. There is a reasonable agreement between both datasets for |*S*_11_|. However, a slight variation in resonant magnitude is noticeable between the measured and simulated data, which may be attributed to several factors such as fabrication tolerances, cable losses, assembly inaccuracies, and the use of bulky end-launch connector. The realized gain plots of the MTM-based series-fed antenna are presented in Fig. 12, showing a comparison between the simulated and measured results. The simulated gain ranges from 11.8 dBi to 15.1 dBi in the desired band of 26.5−40 GHz. The measured gain matches the simulation results for the entire band, with a small discrepancy noted at 35 GHz. This difference can be attributed to the same factors previously mentioned, such as fabrication and assembly inaccuracies and angular misalignment during antenna placement in the anechoic chamber. The far-field measurement procedure has been explained in Fig. 13. The in-house software written in Matlab resides on a dedicated PC machine, and allows for controlling the positions of the reference antenna and device under test (DUT) within a chamber using the step motors. The reference antenna can be rotated with respect to the axis parallel to the ground (to control the polarization), whereas DUT can be rotated with respect to the axis perpendicular to the ground (to measure 2D radiation patterns). The system works in a loop, with antenna positions set by the actuators (with DC supply cut off upon reaching the required rotation angles), and the measurement results acquired from the VNA. The process is continued until accomplishing the entire 2D scan, and the collected results are dumped to the file. In this particular case, we use 0–40 GHz MS4644B Anritsu VNA, and two reference antennas: Geozondas GZ0226DRH (for measurements up to 26.5 GHz), and Pasternack PE9850/2F-15 (for measurements from 26.5 GHz to 40 GHz). The co- and cross-polarization radiation patterns of the MTM antenna in the E- and H-planes at 28 GHz and 38 GHz are displayed in Fig. 14. The developed system offers directional radiation in both planes with a low level of cross-polarized fields. The measured radiation corresponds well with numerical simulation results for the E and H-planes at both frequencies.

The proposed design has been compared to several state-of-the-art antennas reported in the literature, all operating in comparable frequency ranges. The numerical data can be found in Table 1. It is clear that the antenna presented in this study outperforms the benchmark structures with respect to essentially all performance indicators. On the one hand, it exhibits much higher bandwidth covering both 28 GHz and 38 GHz bands. Furthermore, it offers considerably higher gain while being physically smaller by a large margin. On the other hand, it is geometrically simple and straightforward to manufacture within a standard PCB technology.

**Table 1:** Comparison of the proposed antenna with state-of-the-art structures.

Ref	Antenna (λ_0_^3^)	Frequency (GHz)	Bandwidth (GHz)	Max. Gain dBi	Efficiency (%)	Mechanism
^9^	Series-fed dipole (0.93 × 3.4 × 0.019)	28	23.3–41	10.9	–	Eight-element dipole (2D)
^10^	Series-fed dipole (2.17 × 6.36 × 0.048)	28.5	27.25–28.5	10.3	88	Six-element dipole (2D)
^14^	Patch (1.37 × 2.61 × 0.65)	28	27.2–29	13.6	–	Seven MTM layers (3D)
^15^	Patch (1.7 × 2.1 × 1.5)	28	27.1–29.56	11.59	88	MTM layer (3D)
^16^	Bow tie (1.65 × 3.2 × 0.046)	29	26.3–29.8	12.2	92	Etching the MTMs on the substrate (3D)
This work	Series-fed dipole (0.93 × 2.4 × 0.04)	38	26.5–40	11.2	95	Four-element dipole (2D)
	Series-fed + MTMs (0.93 × 3.1 × 0.04)	38	26.5–40	15.1	95.1	Etching the MTMs on the substrate (2D)

## Conclusion

The MTM-based series-fed four-dipole antenna with broad bandwidth, and high gain is reported for 5G mm-wave applications. Although the single dipole covers the 5G band of 28 GHz with a gain of 6.2 dBi, it is still insufficient to compensate for the losses in the mm-wave spectrum. Hence, the series-fed four-dipole configuration is proposed to enhance both the bandwidth and gain. Rigorous numerical optimization is utilized to adjust the dipoles' length and spacing, resulting in an increased gain of 11.2 dBi at 38 GHz. Furthermore, numerical optimization is employed to optimize the MTM dimensions, aiming to achieve a higher refractive index than air within the desired range. As a result, a maximum gain of 15.1 dBi at 38 GHz is attained. The experimentally validated MTM-based series-fed four-dipole antenna demonstrates satisfactory agreement between the simulated and measured data. This design offers a low computational cost, low-profile, and less complex system, along with high gain and broad bandwidth as compared to state-of-the-art developments reported in the literature.

## Data Availability

All Data has been included in study.
